# A space syntax approach to sustainable design for public spaces in historic cities

**DOI:** 10.1371/journal.pone.0351744

**Published:** 2026-07-20

**Authors:** Yanming Zhu, Lue Huang, Cheng Xie, Weiwei Chen

**Affiliations:** 1 South China Agricultural University, Guangzhou, China; 2 Guangzhou College of Applied Science and Technology, Guangzhou, China; Zhejiang Agriculture and Forestry University: Zhejiang A and F University, CHINA

## Abstract

Historic cities, as carriers of urban culture and history, have spatial organizations and usage patterns that directly impact district vitality and sustainability.Addressing the common problems in historic cities of disconnection between public space functions and resident needs, along with insufficient spatial flexibility, this study takes Meizhou Ancient City in Guangdong, China, as an empirical case. It introduces space syntax theory to explore sustainable design pathways. This theory can effectively reveal the strengths and weaknesses of spatial structures. Combined with field research, it constructs data models to quantitatively analyze indicators such as integration, spatial symbiosis, and intelligibility, diagnosing core problems: single-function spaces, weak road network connectivity, and chaotic spatial cognition. Based on this, it proposes a strategy of “Inventory Integration–Functional Reconstruction Interface Optimization”. The results show that after the intervention, the spatial symbiosis and ecological resilience of the ancient city were simultaneously enhanced, contributing to the sustainable development of historic cities. The research validates the scientific supporting role of space syntax theory in the design of public spaces for historic cities. The proposed strategy can effectively buffer disturbances from evolving social demands during the development of historic cities, promoting their sustainable and positive development, and providing a reference paradigm for the renewal of similar commercial-residential mixed historic cities.

## 1. Introduction

### 1.1. Research background

“Piles of rubble line ancient streets and alleys, where culture has been amassed over millennia.” Historic cities constitute not only the core vessels of a city’s collective memory but also key sites where urban history manifests its unique cultural characteristics across time and space [1,2]. Since China established its protection system for historical and cultural cities in 1982, the practice of safeguarding these ancient cities has undergone significant evolution. Initially focusing on protecting individual architectural groups or continuous sections and blocks, the scope of protection has now expanded to encompass comprehensive preservation of the historical district, including its overall appearance and cultural features [3]. Efforts to explore and expand comprehensive preservation are ongoing. As a hub for historical cities, Guangdong Province has recently announced several policy documents such as the “Construction Guidelines for Protection and Enhancement Projects of Historical and Cultural Cities” (Trial Version 2024), “Guangzhou Historical and Cultural City Protection Regulations” (Revised Version 2023), and “Meizhou Historical and Cultural City Protection Regulations” (Revised Version 2023). These documents reflect the government’s attention and determination to preserve historical cities.

Historic cities, as integral components of urban environments, possess distinctive spatial values and cultural significance. However, a disconnection between the functionality of public spaces within these ancient cities and the evolving needs of residents, which is exacerbated by uncertainties, presents significant obstacles to the sustainable modernization development of these historical urban areas [4,5]. Public spaces, serving as essential social hubs and cultural and civic gathering places for residents’ daily lives, are particularly impacted by spatial resilience issues in the current context [6,7]. Therefore, the present study aims to focus on the emerging demands of residents regarding the form and function of public spaces during the development of the historic cities. By employing space syntax as a theoretical framework, we conduct a systematic analysis and assessment of spatial resilience in historic cities to establish targeted optimization strategies and actionable design plans. The objective of the present study is to enhance their adaptability and resilience in meeting the requirements of contemporary societal development while ultimately promoting sustainable evolution within public spaces in historic cities.

Historic cities possess unique spatial value and cultural significance. However, the functional mismatch between public spaces and residents’ evolving needs necessitates analytical methodologies grounded in scientific rigor. As a seminal theory for spatial quantification (Hillier & Hanson, 1984), space syntax employs topological network modeling and visibility graph analysis to systematically decode relationships among spatial connectivity, integration indices, and pedestrian movement prediction. Diverging from urban morphology’s morphological prioritization and new urbanism’s sociocultural prescriptions, space syntax leverages mathematical graph theory to mitigate methodological subjectivity. Its analytical framework enables precise quantification of heritage districts’ spatial permeability, while Depthmap-derived intelligibility metrics (Jiang & Claramunt, 2002) effectively forecast pedestrian flow patterns. This capacity to operationalize spatial self-organization principles provides critical solutions to reconciling quantitative compatibility between cultural preservation imperatives and functional modernization demands.

In the study of enhancing spatial resilience in historic cities, the applicability of space syntax can be firmly established through both theoretical reasoning and empirical validation. Theoretically, urban spatial resilience fundamentally entails the capacity of a spatial system to maintain or rapidly restore its core functions when confronted with disruptions [8]. Space syntax quantitatively characterizes topological relationships among spatial elements using core metrics such as integration, connectivity, and mean depth, thereby providing an operational morphological dimension for assessing such capacity. Integration corresponds to the efficiency of resource allocation and the inclusivity of activities in resilience evaluation—spaces with high integration can respond swiftly to diverse demands and mitigate risks of functional interruptions. Connectivity reflects the redundancy of the spatial network; higher redundancy allows functions to be maintained through alternative pathways, aligning with disturbance resistance and recovery capabilities. Mean depth indicates the level of spatial permeability, where excessively high values may form critical vulnerabilities in resilience and are directly linked to operational stability [9]. Empirically, a case study of the historic district along West Nanjing Road in Shanghai demonstrates a significant correlation between space syntax indicators and resilience performance. An increase in integration led to a marked improvement in evacuation efficiency during sudden surges in pedestrian flow, while connectivity optimization substantially reduced the risk of functional breakdown [10]. Research in the historic district of Yushan, China, further corroborates that streets with low integration and connectivity (e.g., areas dominated by dead-end alleys) exhibit significantly lower survival rates of commercial functions compared to highly integrated streets, identifying them as key areas requiring intervention to enhance resilience [9]. Thus, space syntax enables the precise identification of morphological deficiencies—such as high density of cul-de-sacs and functional homogeneity—that undermine resilience in historic cities. Furthermore, employing axial models and visibility analysis within space syntax can elucidate deeper structural issues in spatial configuration.The quantitative analysis framework of spatial syntax not only enhances the scientificity and operability of the strategy, but also realizes the effective connection from spatial structure analysis to sustainable development of the ancient city.

### 1.2. Research significance

This study investigates sustainable design approaches for public spaces in Historic cities through the lens of space syntax theory. Conventional preservation and revitalization practices have often emphasized physical renovations while overlooking holistic and systematic integrity. In contrast, the concept of spatial resilience extends beyond this narrow focus by examining the comprehensive performance of spatial organizations. It promotes a shift from isolated physical interventions toward integrated and systematic optimization, offering both theoretical and practical implications for broadening the scope of historic district research.

The rapid growth in tourism has significantly increased visitor numbers in many historic cities, disrupting the daily lives of local residents and limiting their activities mainly to residential and private areas. At the same time, existing public spaces increasingly fail to meet residents’ emerging social needs. These challenges underscore the urgency of enhancing the sustainability of public spaces in historic urban settings to accommodate diverse and evolving demands. Using Meizhou, Guangdong—a typical mixed-use historic district—as a case study, this research develops an analytical model and proposes revitalization strategies that can serve as a valuable reference for similar preservation and regeneration projects.

## 2. Literature review

### 2.1. Research situation

After four decades of domestic practices and explorations in the protection of historic cities since 1979, a comprehensive theoretical framework for conservation has been established, incorporating multidisciplinary and multi-perspective approaches. For instance, Xu (2015) applied the theory of symbiosis in the renovation design of Dalian’s Dongguan Street, providing guidance for revitalizing historical areas through symbiotic environments, units, and systems [11]. Furthermore, various theories and methods such as micro-renewal techniques, scenography principles, and generative theories have been extensively utilized in conserving and renewing historic cities.As noted by Shi (2020), research on conserving and revitalizing historic cities spans a broad and intricate scope [12]. For instance, Liu (2015) conducted an in-depth investigation into the protection and diachronic reshaping of original resources and values in Fuzhou’s Zhuzifang historical district, focusing on the evolution of its spatial form [13]. Yang (2019) established a theoretical framework to analyze the socio-cultural and spatial planning mechanisms of integrating modern elements into historic cities, with a focus on resolving conflicts between heritage preservation and contemporary urban demands [14]. Duan (2018) proposed a community-driven approach to revitalize historical street areas, emphasizing resident participation and cultural continuity as key strategies for enhancing both physical infrastructure and social vitality [15]. Furthermore, Wu (2020) comprehensively explored the revival of regional culture through considering multiple factors such as culture, industry, and architecture in the conservation and renewal design of Shouzhou ancient city’s historical district [16]. With the widespread application of digital technology and the diversification of research tools, Chen (2009) conducted a comprehensive quantitative study on the spatial form of Fuzhou’s Three Lanes and Seven Alleys using space syntax theory, encompassing urban planning, district analysis, and architectural evaluation. His meticulous investigation not only established a robust theoretical foundation for the conservation, revitalization, and rejuvenation of this area but also offered invaluable practical guidance [17].

In recent years, international scholarly research on the resilience of public spaces in historic cities, grounded in space syntax theory, has progressively deepened. The methodological application of this theory across diverse cultural contexts offers critical insights for the conservation of ancient cities in China. For instance, studies on Rome’s historic center demonstrate that optimizing public space networks through circular economy strategies significantly enhances the adaptive capacity of ancient urban areas to withstand tourism pressures and sudden disruptions (Fusco Girard, 2020). In Athens, scholars employed space syntax to quantify pedestrian accessibility and visual permeability in districts surrounding the Acropolis, revealing topological conflicts between historical axes and modern transportation networks. This analysis informed proposals to restore fragmented spatial connections via micro-regeneration strategies (Chatziioannou, 2021). Empirical research in Istanbul further validates the applicability of space syntax: addressing the “island effect” in the historical peninsula, scholars advocated for strengthening functional hybridity by activating topological linkages in secondary alleys, thereby improving community resilience to risks (Yıldırım, 2022). The revitalization of Paris’s Marais District exemplifies a balanced approach to heritage preservation and functional renewal—by enhancing the intelligibility of public spaces and interface continuity, the district retains its historical character while integrating modern amenities (Gravari-Barbas, 2018). Additionally, a global study by van Nes and Yamu (2021) systematically substantiates the universality of space syntax in assessing historic urban resilience, identifying functional redundancy and topological integration as key predictors of spatial resistance to disturbances. These findings collectively indicate that space syntax not only deciphers structural contradictions in historic cities but also establishes a cross-cultural, multi-scalar design framework for resilience enhancement through quantitative analysis. This approach addresses the limitations of traditional qualitative research by providing robust data support and strategic precision.

In the study of revitalizing ancient cities in China and abroad, prior research has mainly concentrated on qualitative factors like cultural continuity, sense of localization, and cognitive perceptions. However, these studies often lack scientific rationale and universal applicability. It is necessary to identify scientific theories that are suitable for historic cities in order to address the limitations of traditional subjective and experiential research. Identifying space syntax theory will inject more objective and rigorous scientific analysis into the optimization of public spaces in historic cities. By utilizing quantitative analysis with its high operability and strong argumentative power, robust data support can be provided for developing renewal strategies. Spatial syntax can effectively achieve it. These new strategies will drive a methodological shift in public space optimization research for historic cities from perception-based qualitative studies towards a more scientific, quantitative, professional, and systematic paradigm [4,18,19].

### 2.2. Objects

This study focuses on the public spaces of the historical ancient city in Meizhou, Guangdong Province. Due to its unique cultural and historical characteristics, it holds significant research value. As a national historical and cultural city with a thousand-year cultural heritage, Meizhou Ancient City not only preserves a relatively complete public space layout but also carries rich Hakka cultural heritage and distinctive architectural styles. These characteristics make it an ideal subject for studying traditional urban structures, the evolution of historic cities, and regional cultural inheritance.

Specifically, the public space of the research object refers to the core historical district of Meizhou Ancient City, defined as the space carriers with open and shared attributes and bearing core public functions such as public communication and passage, mainly including public streets, squares, and open public courtyards, etc. These spaces are not only important venues for social activities but also key carriers of cultural inheritance and urban vitality.

The research scope mainly focuses on the urban area of Meizhou Ancient City.

**Fig 1:**
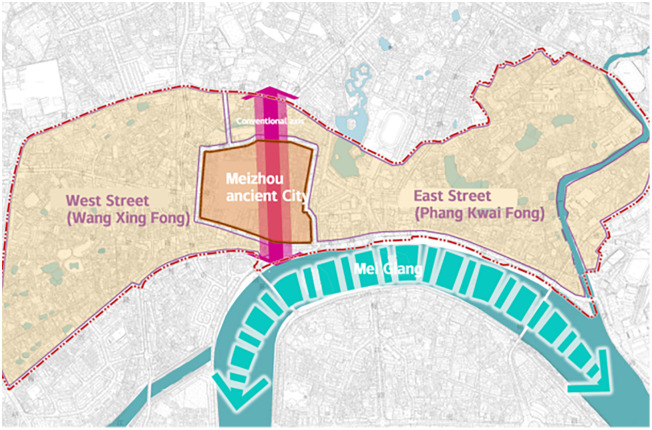
Location map of Meizhou Ancient City.

This area is composed of three historic cities, namely Lingfeng East and West Roads, Zhongshan Road, and Jinshazheng Road. As outlined in the “Meizhou Historical and Cultural City Conservation Plan (2015-2030),” a research area spanning 15,900 square kilometers has been designated for conservation purposes depicted in Fig 1, with particular emphasis placed on the historical district covering 2.64 square kilometers. Situated in the central-western part of Meizhou, Meizhou Ancient City is bounded by the Mei River to the south, Meizhou Avenue to the north, Zhongshan Road to the west, and Taikang Road to the east. It serves as an illustrious urban center that encapsulates both the abundant local cultural heritages and distinctive architectural characters unique to this region. There are a total of six cultural relic protection sites within the ancient city, including three at the municipal level and three at the county level. Among them, the Arcade-style Traditional Commercial Streets of Lingfeng East-West Road, the Huang Family’s Ancestral Hall, and the Meizhou Confucian Temple have been designated as municipal-level protected units. Additionally, there are seven immovable cultural relics that have yet to receive official recognition as protected units, along with numerous buildings showcasing traditional styles.

These invaluable cultural architectural heritages not only embody the developmental history, local customs, and profound Hakka cultural traditions of the Meizhou region but also serve as iconic representations of its regional architectural style. They constitute an indispensable core element and a focal point for conservation and revitalization endeavors within Meizhou Ancient City. Through in-depth investigations and studies on the preservation of architectural styles, the protection of cultural heritage, and the functional positioning of public spaces, the unique value of the Meizhou Ancient City has been more fully demonstrated.

**Fig 2:**
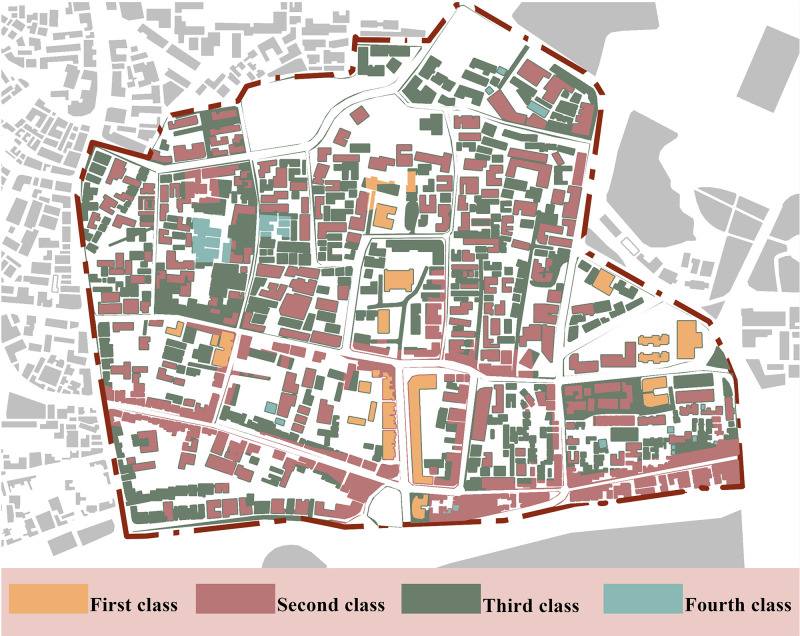
Building quality table of Meizhou ancient city.

**Fig 3:**
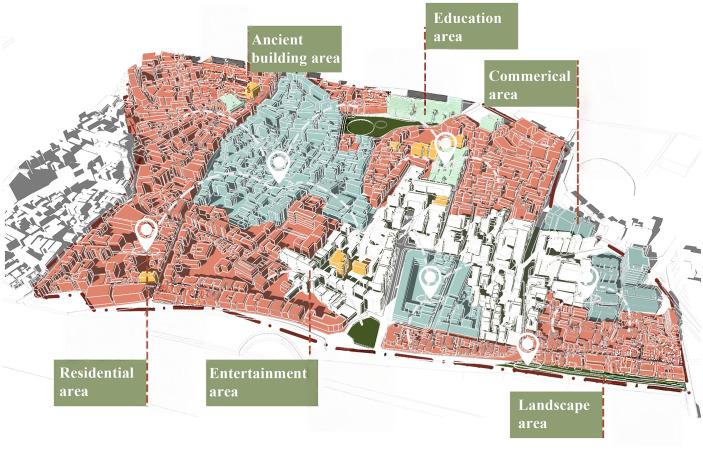
The current spatial function status of Meizhou ancient city.

Fig 2 presents a systematic review of the relevant data on the building quality within Meizhou Ancient City and a field investigation. It shows that half of the buildings in the ancient city area have a quality of grade three or four, which is considered poor. Additionally, the fragmentation of the functional groups in the ancient city has led to an “island effect” between the groups as depicted in Fig 3. The above research data on the characteristics of the quality of buildings and the fragmentation of spatial structure within Meizhou Ancient City provide crucial support for the application of the quantitative analysis tool of spatial syntax in the study of Meizhou Ancient City, and lay the foundation for subsequent research on the classification of building quality and spatial optimization.

Based on the first-hand data obtained from the on-site investigation, such as the preservation status of the main building structure, the construction era, and the maintenance history, this study has constructed a three-dimensional evaluation index system of “structural stability - construction process integrity - usage safety”. Combined with the actual characteristics of the historical buildings in Meizhou, the building quality is classified into four grades: Grade 1 (quality) buildings refer to those with good structural stability, no obvious damage to the construction process and appearance style, and a solid main structure; Grade 2 (quality) buildings refer to those with good structural stability, a complete overall structure but with slight damage to the plaster layer of the walls and doors and windows; Grade 3 (quality) buildings refer to those with poor structural stability, with different degrees of erosion and damage to the main structure (walls, roofs), being in a state of long-term disrepair, and having safety hazards; Grade 4 (quality) buildings refer to those with poor structural stability, severe damage to the roof, the main structure is on the verge of collapse, and there are serious safety hazards, and should be demolished and rebuilt.

Although this study uses spatial syntax as a quantitative analysis tool to analyze the spatial form and architectural protection status of ancient cities, we cannot ignore the importance of qualitative research in this field.It can delve into the cultural, social, and historical contexts that shape the identity of ancient cities and the emotional connections between communities and spaces. Therefore, Combining quantitative data with qualitative insights can provide a more comprehensive understanding of the revitalization of ancient cities.

### 2.3. Innovation

Spatial syntax provides a powerful tool for analyzing how spatial layouts affect the vitality and sustainability of public spaces in ancient cities. Driven by rapid changes in tourism development and social demands, many historic cities are facing challenges such as the decline of public space functions and the deterioration of livability, and urgently need to be guided by sustainable design thinking to determine their regeneration paths. This study takes the historical ancient city of Meizhou in Guangdong Province as a case, builds an assessment model based on spatial syntax, quantitatively analyzes core spatial elements such as integration and connectivity, systematically diagnoses the spatial resilience level of the district, and provides a basis for formulating sustainable regeneration strategies that balance social vitality, cultural continuity, and environmental adaptability.

Research shows that the urban spatial structure profoundly shapes the possibilities of public life: highly integrated and easily understandable street networks not only enhance pedestrian accessibility and human-scale experiences, but also effectively promote informal social interactions and strengthen community cohesion. This highly permeable and open spatial pattern provides a dailyized carrier for local cultural practices, enabling traditional festivals, markets, and neighborhood interactions to continue to thrive in contemporary urban environments. Therefore, integrating spatial syntax into the renewal design of historic cities helps to retain the material texture while activating its function as a social and cultural container, promoting truly inclusive, resilient, and sustainable urban regeneration.

## 3. Methods

The research team systematically collected and organized the literature related to the Meizhou Historic Area, explored the historical background, current development, theoretical views and future trends of the area, and conducted in-depth analysis and interpretation of the existing literature to comprehensively understand the development of the Meizhou Historic Area and the evolution of its spatial patterns. Based on the research objectives, the research team comprehensively adopted a variety of methods such as photo recording and on-site observation to conduct field investigations and visits to the Meizhou Historic Area and its surrounding areas, recording in detail key information including the spatial distribution of functions, the accessibility of streets and alleys, and the spatial constituent elements of the area. The status of alleys closed by fences, as well as buildings that have been added or demolished, was identified as a basis for a more intuitive understanding of the current public space situation in the Meizhou Ancient City.

**Fig 4:**
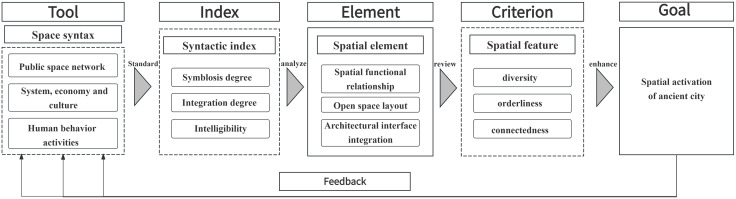
Construction of space syntax evaluation for historic cities.

Based on the site self-construction-area maps obtained from the field research, an exhaustive spatial model of the historical area of Meizhou is constructed in Fig 4. By using the professional analysis software in the principle of space syntax, the public space of the historical area was transformed into an accurate axial model, selecting the three-dimensional space within a specific range and picking out the axes, concise buildings and spatial relations that can run through the whole area, transforming them into the axial model, and analyzing them through the axial line and the scattering map to show the interactive relationship between these spatial units. Numerical analysis was carried out with the help of the Depthmap program to conduct a quantitative study, combining the various conservation evaluation indicators of the historical area and qualitative factors such as the motivational needs of the personnel, in order to ensure the objectivity and scientificity of the results of the study, providing a strong data for the study as depicted in Fig 4.

The present study took the public space of Meizhou Ancient City as the research project, translated the external morphological elements of the public space of the ancient city into urban morphology, and used the space syntax analysis method to quantify the external morphological elements of the space, analyzed the mutual influence of the spatial morphological elements and people’s behavioral activities. the present study also aimed to explore the methods to enhance the diversity of the functions of the public space of the ancient city, optimize the network structure of the ancient city streets and lanes and revitalize the public space of the ancient city to better adapt to the current sustainable development of cities and the needs of residents’ lives.

## 4. Analysis process

### 4.1. Analysis of spatial integration and connectivity

This study is based on the “triple bottom line” framework of sustainable design (Elkington, 1997), and examines the spatial structure of historical districts from three dimensions: environment, society, and economy [20]. Among them, social sustainability is particularly crucial, emphasizing the accessibility, inclusiveness, and cultural vitality of public spaces. The distribution of integration degree revealed by spatial syntax serves as an important quantitative basis for evaluating these attributes.

**Fig 5:**
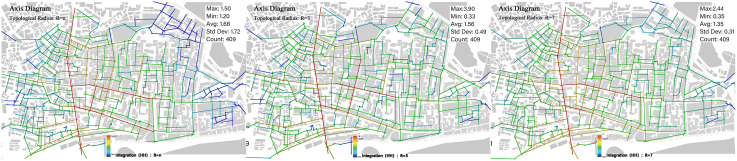
Integration of Meizhou Ancient City.

The measure of integration is determined by the number of connections between a unit space axis and other unit space axes. This metric aims to evaluate the accessibility of a unit space, quantifying its connectivity and ease of access. Integration can be categorized into two types: global integration and regional integration. Global integration indicates enhanced spatial openness and permeability, attracting frequent usage from larger crowds [21]. Regional integration reflects the degree of connectivity between a unit space and its neighboring spaces within various topological distance ranges. The intensity of darker red color on the axis in Fig 5 represents higher connectivity, suggesting more frequent activities and interactions within the space, as well as greater spatial vitality with stronger influence on the surrounding environment. Conversely, lighter colors indicate lower connectivity and vitality [22].

Selecting 409 effective axes in Meizhou Ancient City, the calculation legend demonstrated that: Firstly, areas with high integration are predominantly located on the eastern side of the area. The red axes namely Zhongshan Road, Zhongyuan West Road, and Lingfeng West Road serve as primary thoroughfares within the city. Syntax analysis revealed their significant accessibility advantages, showing them as key spatial nodes for pedestrians and vehicles within the block. Among these axes, Zhongshan Road stands out prominently due to its darker tone (color) representation, indicating exceptional public accessibility. Undoubtedly, this road serves as a vibrant social activity hub for the entire historical area in Fig 5. Secondly, upon analyzing the distribution of roads with high integration values, it is observed that streets positioned at either edge or center of the ancient city exhibit higher values, in Table 1 while color intensity diminishes noticeably on the eastern side of the ancient city. This discrepancy suggested a substantial gap in integration values between both sides and highlighted a weak correlation along with limited crowd flow potential. Consequently, the discrepancy became less favorable for generating social activities and economic benefits. Furthermore, several axes connected to Zhongshan Road displayed significant differences in integration values signifying relatively monotonous functions on either side of these streets lacking diverse space utilization in Table 2 and positive interaction. These findings indicate an uneven distribution of vitality within Meizhou Ancient City which hampers balanced development.

**Table 1:** Integration of Meizhou Ancient City.

value	Max	Min	Avg	Std Dev	Count
Global Integration (R = n)	1.20	1.50	1.68	1.72	409
Regional integration (R = 3)	3.90	0.33	1.56	0.49	409
Regional integration (R = 7)	2.44	0.35	1.35	0.31	409

**Table 2:** Integration of main roads in Meizhou Ancient City.

Road Name	Lingfeng East Road	Lingfeng West Road	Zhongyuan East Road	Zhongshan Road	Jinshan Road
Global Integration	1.20	1.50	1.68	1.72	1.10

The quantification of Meizhou Ancient City’s integration revealed stark contrasts in the colors denoting levels of integration, indicating poor functional connectivity. Field visits and investigations demonstrated weak connections among the ancient city’s spatial, architectural, and humanistic functions, primarily manifested through a gradual weakening of structural links between historical spatial elements such as center, boundary, and axis. In the process of rapid urbanization, the original spatial pattern of the ancient city struggled to adapt to the spatial transformation needs imposed by modern functions due to “space-time compression.” Consequently, this has resulted in fragmentation into spaces of varying sizes within what was once a cohesive ancient city. Simultaneously, numerous abandoned spaces and blocked laneways had further intensified the division among architectural spatial function groups, forming a pattern composed of fragmented groups and small functional areas. Architectural relics are scattered throughout these blocks with existing closed-circle protection neglecting proper transition and connection between inner and outer spaces within these areas. This type of conservation approach may lead to deep burial of buildings creating an “island” effect that hampers recognition of the overall image of the ancient city. At a humanistic space level, weak connectivity is reflected in gradually diminishing functional links between historical blocks and surrounding communities concerning business complementarity, facility sharing, and social communication. As a result, residents may struggle to integrate into broader community life areas within the ancient city while weak connectivity ultimately undermines its functional resilience and vitality setting off a negative cycle.

### 4.2. Analysis of symbiosis and spatial function diversity

**Fig 6:**
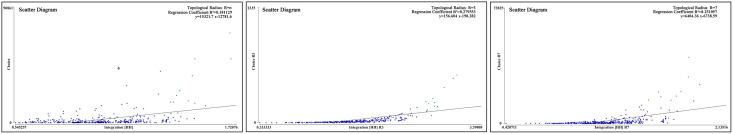
Spatial symbiosis of Meizhou ancient city.

The presence of symbiosis is demonstrated through direct linear associations across various levels of integration and screening, highlighting the robustness of functional composition within specific regional spaces. In the XY distribution graph in Fig 6, the vertical axis represents the selection index, while the horizontal axis denotes the integration index. The symbiosis index is indicated by the R^2^ value. X signifies the degree of incorporation, whereas Y represents the degree of selection. The coefficient of determination (R^2^) in its linear regression quantitatively characterizes the mixed attributes of regional space. Symbiosis refers to a spatial capacity for accommodating multiple functions. An R^2^, as a square measure known as topological radius R, defines an area influenced by function: higher numerical values indicate stronger abilities to incorporate functions; conversely, lower numerical values suggest relatively limited function-carrying capacities [23].

The spatial structure distribution maps of Meizhou Ancient City under different topological radii, namely R = n, R = 3, and R = 7 in Fig 6, are presented below. The specific values of the symbiosis equation and its coefficient of determination R^2^ can be found in Fig 6 as well. Analysis and calculations revealed a decrease in symbiosis value with the expansion of the inspection boundary. Moreover, the generally low symbiosis indexes indicated a lack of functional diversity within Meizhou Ancient City.This single-functionality not only limits the flexibility of space conversion, but also weakens the community’s ability to respond to emergencies, violating the principle of functional redundancy and flexibility emphasized in sustainable design.

The field investigation data on Meizhou Ancient City also confirms that this ancient city exhibited a combination of commercial and residential functions. However, the commercial activities primarily revolved around street catering and daily hardware facilities, indicating a noticeable concentration of functional land use. Notably, the city lacks functional diversity as evidenced by a scarcity of community service facilities and venues for entertainment and cultural activities. The primary focus in the life of this ancient city is to achieve self-sufficiency in terms of functionality, and ensure that residents have access to comprehensive supporting facilities within walking distance while being equipped to handle emergencies effectively. Nevertheless, the current state of the ancient city fell short in meeting residents’ diverse needs related to safety, daily activities, and other aspects due to an absence of spatial structures with functional redundancy. Not only was there insufficient reserved emergency backup space, but the rigid functional zoning also strictly limited various land uses to a single purpose, hindering the rapid adjustment of spatial functions. This lack of functional redundancy directly limits the flexibility of space transformation – for instance, in the event of an emergency, there is no idle space available for quick conversion into temporary shelters, distribution points, or medical aid stations. Moreover, the rigid commercial and residential zoning along the street cannot be quickly transformed into emergency service functions.Consequently, Meizhou Ancient City struggled to expeditiously adapt normal functions into emergency functions when faced with unforeseen disturbances, significantly undermining the resilience and adaptability of public spaces following such disruptions. This deficiency may reduce space utilization efficiency while increasing vulnerability towards spatial functionalities.

In conclusion, enhancing the spatial functional diversity and symbiotic relationship of the Meizhou Ancient City is one of the key paths to achieving sustainable regeneration. By optimizing the spatial layout and increasing functional redundancy, the social inclusiveness, environmental adaptability and economic vitality of the ancient city can be significantly improved, thereby promoting more comprehensive and resilient sustainable development.

### 4.3. Analysis of spatial intelligibility and cognitive perception

**Fig 7:**
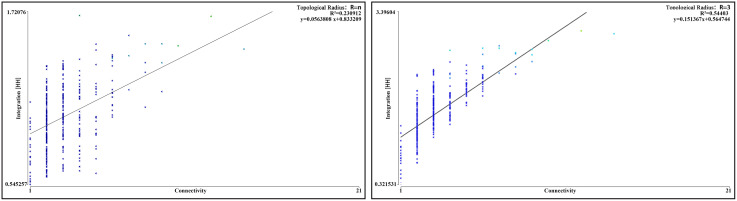
Intelligibility of Meizhou ancient city.

Intelligibility, also known as intelligent comprehensibility, measures the ease with which a spatial structure can be recognized. The intelligibility index is determined by the numerical ratio of overall space integration to connectivity [24]. In the XY plane that describes intelligibility, the X-axis represents the overall integration of space, while the Y-axis denotes its overall connectivity in Fig 7. The value R^2^ characterizes intelligibility numerically. An R^2^ value below 0.5 indicates low intelligibility of spatial structure; between 0.5 and 0.7 suggests medium intelligibility; above 0.7 implies high intelligibility, making the spatial layout more discernible. Moreover, evaluating residents’ ability to predict the overall spatial layout based on their understanding of local spaces is interpreted by the R^2^ value [21,25,26].

The calculated overall and local cognition indexes of Meizhou Ancient City and its adjacent areas, as presented in the charts in Fig 7, demonstrated fitted R^2^ values between 0.230 and 0.544. These findings indicated a relatively higher level of local intelligibility within the block but a lack of global intelligibility. Due to the intricate spatial layout, residents walking or driving on the main roads may experience difficulties in accurately perceiving directions. However, within the walkable confines of the topological structure, people’s predictions about the surrounding spatial layout tended to be more accurate, reducing disorientation likelihood. This phenomenon primarily arised from the complex interweaving of roads in both Meizhou Ancient City and its vicinity, compounded by insufficient route guidance and a lack of orderliness. Moreover, variations in spatial connections between different junctions at street entrances can easily lead to visual misguidance that affects individuals’ spatial cognition and sense of direction while diminishing overall recognizability within this area. To address these challenges effectively, activation designs should place special emphasis on enhancing connectivity and directional guidance along main streets while improving traffic functions through clear signage implementation and zoning enhancements.

The analysis of intelligibility in Meizhou Ancient City’s public spaces revealed a score below 0.5, indicating a limited understanding among individuals regarding the architectural interfaces and potential confusion. Moreover, field investigations confirmed that the visual perception of architectural interfaces aligns with these calculations. While numerous arcade building groups existed within the city, Lingfeng East Road stands as a well-preserved municipal heritage conservation site in this regard. However, this street was marred by cluttered low-end small shops, haphazardly installed medium and small-sized electrical boxes, disorderly distributed street signs, and illegally parked cars. These issues significantly disrupt the continuity of the visual space, eroding not only the appearance but also posing safety hazards to these historic streets. Additionally, alleyways and residential entrances served as active mixed-interface spaces within this street interface; however, there was currently a stark fragmentation of these interfaces in the area with notable loss of sense of place at Lingfeng West Road and Zhongshan Road junctions adversely affecting residents’ utilization of street space. Furthermore, disarray in architectural and street interfaces may lead to confusion over direction for both residents and visitors alike while navigating through Meizhou Ancient City resulting in potential safety risks.

## 5. Results and discussion

### 5.1. Recommendations

#### 5.1.1. Integration and optimization of inventory space.

Through the spatial syntax analysis method, an integrated study of the Meizhou Ancient City was conducted, revealing that areas with low integration potential possess the potential for improving spatial quality. Specifically, by demolishing illegal buildings, dilapidated houses, and structures with low protection value, public spaces that have been occupied or privatized can be released. Such renovations should be based on the core principle of respecting and preserving the original architectural texture, avoiding the destruction of the spatial context of the historical district.

**Fig 8:**
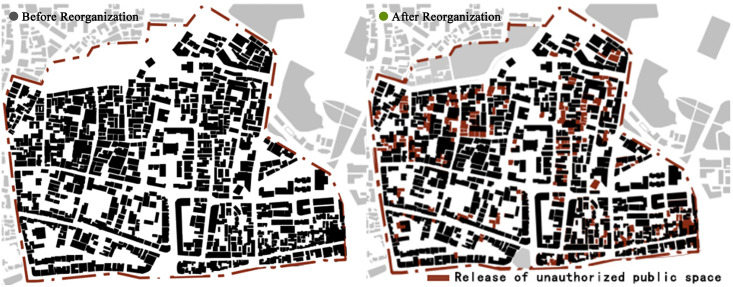
Comparison of public spaces in Meizhou ancient city before and after renovation. Before Reorganization: Illegal constructions in some areas closely connected with buildings. After Reorganization: Existing public spaces expanded, integrating small spaces.

Although the internal spatial form and texture of the Meizhou Ancient City present a high degree of diversity, its overall layout has significantly improved in terms of organization and visibility after the intervention of demolition. As shown in Fig 8, the proportion of public spaces has increased significantly, providing more flexible and diverse possibilities for the future layout of parking lots, community activity areas, pedestrian paths, and central squares, thus laying the foundation for sustainable design integration.

This strategy has been supported by successful practical examples. For instance, the micro-renewal project of Yangmeizhuo Slant Street (Dagashan Area) in Beijing strictly preserved the original hutong texture. It demolished illegal constructions and reclaimed inefficient commercial spaces, thereby integrating scattered ‘corner areas’ into composite functional nodes such as mini pocket parks, community libraries, and shared courtyards.The project did not rely on road widening but improved accessibility and daily vitality through refined spatial reorganization. The result not only increased residents’ satisfaction but also promoted the organic integration of cultural tourism and local life, effectively avoiding “gentrification” due to excessive commercialization and the loss of local residents, achieving sustainable regeneration in the social, cultural, and environmental dimensions.

#### 5.1.2. Diversification of spatial functions.

The “Culture Reservoir Base” in Seoul provides an extremely inspiring example for the functional regeneration of historical spaces. This project transformed a long-abandoned water storage tank into a multi-functional cultural complex that integrates art exhibitions, community workshops, family-friendly play areas, ecological education, and temporary markets. This space effectively meets the diverse needs of residents, tourists, and artists, not only revitalizing the dormant urban assets but also becoming a “third space” with inclusiveness and self-organizing capabilities, significantly enhancing the social value and sustainable operational potential of public spaces.

**Fig 9:**
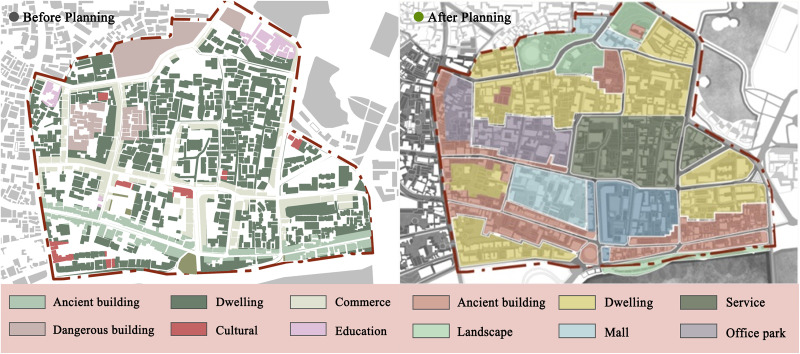
Comparison of functional layout of Meizhou ancient city before and after planning. Before Planning: Limited functions with a low and fragmented distribution. After Planning: Diverse functions with integrated zoning.

This practice demonstrates that the sustainability of historical spaces does not stem from static preservation, but rather from building a virtuous cycle through functional diversification. Inspired by this, this study, based on the symbiotic relationship analysis of the spatial syntax of the Meizhou Ancient City, identified several areas with low symbiotic value and proposed targeted functional integration strategies: By introducing new economic and cultural leadership functions are introduced to fulfill the developmental needs of the ancient city. Furthermore, surveys on human contextual needs revealed an increased demand among residents and tourists for auxiliary leisure and entertainment functions. Therefore, in planning and designing, while ensuring the protection of existing historical features, there should be an emphasis on enhancing the diversity of block functions and the variety of supporting facilities as proposed in Fig 9. This is a key link in achieving sustainable use of public spaces.

#### 5.1.3. Optimization of the street-facing commercial interface.

The interface update of the arcade street in Guangzhou Yongqingfang provides an experience reference for the transformation of the area into a historical district of Lingnan culture. Yongqingfang achieved the integration of the old and the new by implementing a unified guideline – namely, uniform eaves height, window-to-door ratio, and base color. At the same time, it allowed for personalized display windows, distinctive signs, and green plant decorations, while preserving traditional elements such as Manchu windows and brick carvings. This strategy not only maintained the historical continuity of Lingnan arcades but also brought vitality to the area through the expression of individual store characteristics. The street interface is not only visually harmonious but also shows rich dynamic changes, enhancing the overall walking experience and the appeal of public spaces.

**Fig 10:**
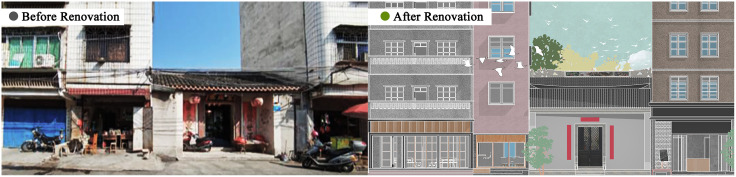
Comparison of architectural interface of Meizhou ancient city before and after renovation. Before Renovation: Disorganized architectural interface and exposed wiring bringing safety hazards and poor scenery. After Renovation: Uniform material use across varied business types for cohesive architectural interfaces with distinct styles.

Inspired by this,based on the intelligibility analysis constructed using the space syntax for Meizhou Ancient City, there was a large number of arcade buildings within the ancient city. The municipal heritage conservation buildings on Lingfeng East Road were well-maintained in terms of architectural interface, warranting a protective approach. In contrast, the shops on Zhongshan Road and Jinshanding Road were in poor condition and lack stylistic coherence. For storefronts serving various functions, a consistent material was used in their design to maintain visual unity. However, each storefront employed distinctive design techniques and themes, crafting a harmonious but contrasting effect that enhances the visual dynamics and, consequently, the rhythm and appeal of the street interface. This strategic approach in design promoted a sense of continuity and flow in the spatial interface, encouraging pedestrians to continue their journey and improving the overall walking experience. It enhanced the awareness and vitality of the space, reflecting the sustainable design concept of “beauty and harmony in diversity” in Fig 10.

### 5.2 Assessment

#### 5.2.1 Improvement of street network connectivity in historical areas.

(1) Breaking through the ownership of street boundaries

**Fig 11:**
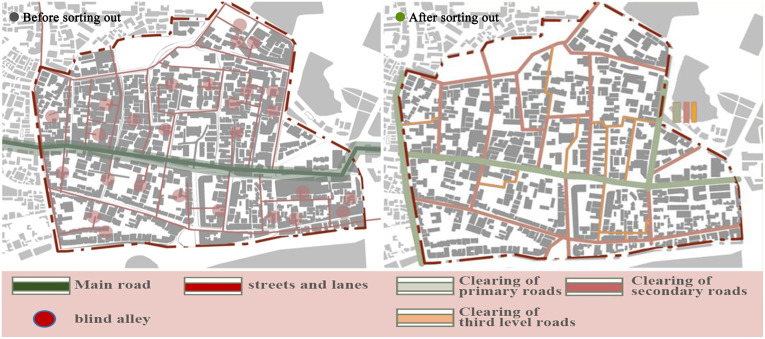
Comparison of streets and alleys in Meizhou historical area before and after sorting. Before sorting out: There are a large number of “dead end roads” in the historical area, with roads cut off and spaces fragmented. After sorting out: According to the street scale and functional attributes, while clearing the road, divide it into three levels according to the standard.

Improving the connectivity of the street network is a key sustainable design strategy for historic cities to achieve social inclusiveness, pedestrian friendliness and spatial resilience.According to the spatial syntax analysis of Meizhou’s historical area, the street and alley system is characterized by low accessibility and convenience, with numerous “dead-end roads.” To address this issue, three strategies are proposed. First, dredging measures should be implemented to improve dead-end roads and underutilized spaces, based on the integration and optimization of existing spatial resources. Second, leveraging the current road network structure, a systematic reorganization and hierarchical classification of block roads should be carried out to establish a three-level traffic system. This would involve moderately increasing road network density to enhance internal spatial connectivity and integration. Finally, physical barriers such as high enclosure walls can be lowered or replaced with semi-transparent fences to expand visual boundaries and better meet residents’ needs for communication and visual openness within the block (Fig 11).

(2) Optimize street traffic routes

**Fig 12:**
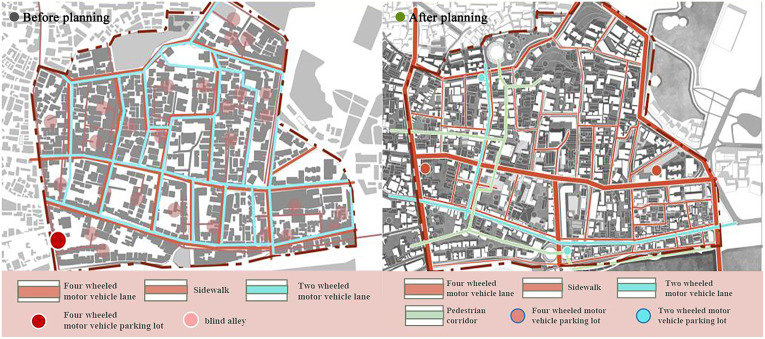
Comparison before and after traffic sorting in Meizhou historical area. Before planning: The traffic in the historical area is mixed, and vehicles are parked randomly occupying the road. After planning: Stratified diversion based on road size and vehicle function, with newly added non motorized parking spaces for motor vehicles.

Optimizing the traffic organization of the streets is the core approach to achieving sustainable design goals such as low-carbon travel, human-scale design, and functional integration in historic cities.To address the issue of mixed traffic involving various vehicle types in the narrow neighborhoods of Meizhou’s historical district, three strategies are proposed. First, the existing road space should be systematically organized, with road functions classified according to user needs, and traffic routes planned based on street scale to reduce pressure on smaller alleys. Second, while retaining the original layout, new roads can be constructed, and pedestrian walkways and landscape corridors introduced to disperse foot traffic and provide diverse movement options. Finally, by flexibly reorganizing the existing space and setting up appropriately located parking facilities for both motorized and non-motorized vehicles, the traffic burden on the street network can be significantly alleviated (Fig 12).

#### 5.2.2. Rich and improved ecological space in historical areas.

(1) Diversity of landscape types

**Fig 13:**
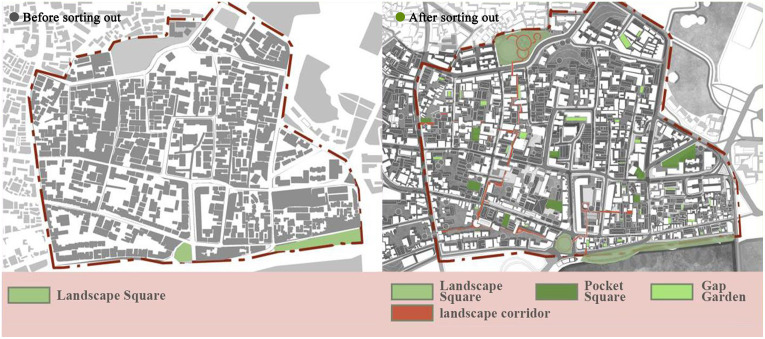
Comparison of landscape types in Meizhou Historical Area before and after. Before sorting out: Lack of landscape space within the historical area. After sorting out: Design different landscape spaces according to different spatial scales to meet the needs of different populations.

The historical area of Meizhou lacks open public spaces, prompting the addition of activity spaces to better meet residents’ needs. First, public spaces with high visual integration and strong safety are selected, where landscape forms are adapted to the needs of different local populations. Large-scale landscape squares and small-scale pocket parks are then planned accordingly. In areas not covered by these main spaces, small-scale landscape installations are introduced to create unique crevice gardens, effectively fulfilling the diverse activity space needs of various community groups (Fig 13).

(2) Building a landscape corridor

**Fig 14:**
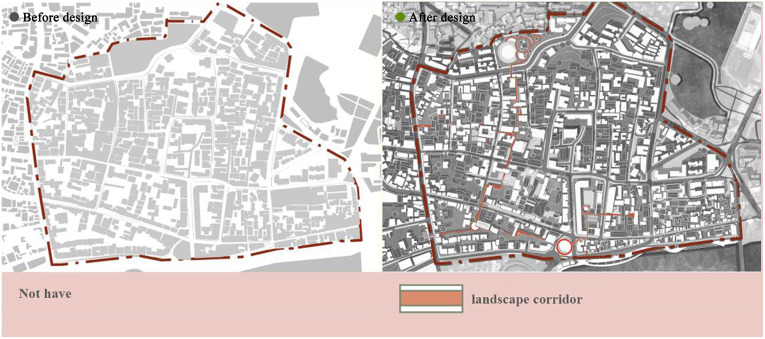
Comparison of landscape corridor design before and after Meizhou historical area. Before design: No separate trail system. After design: Improve the trail system and landscape network of the landscape corridor.

In Meizhou’s historical area, a convenient, accessible, and open trail system has been established to reconnect fragmented landscape belts, stimulate resident activities, and enhance the vitality of green spaces. An accessible and visually engaging circulation system has been created, with landscape squares, cultural and sports facilities, and scattered residential areas serving as key nodes. In Fig 14, these nodes are linked linearly to form corridors that support pedestrian movement, leisure walking, and recreational activities. Ecological bridges connect urban “islands” formed by fragmented green spaces, enhancing interaction between people and the environment, and improving the connectivity and stability of the overall urban ecological network.

(3) Emerging technologies for greening

**Fig 15:**
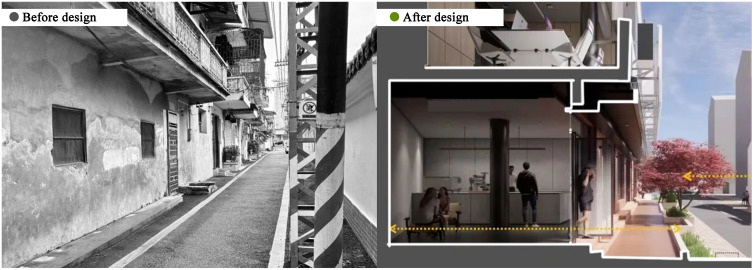
Comparison table of streets in Meizhou historical area before and after renovation. Before planning: The ground pavement in the historical area is asphalt road, which is not conducive to drainage. After planning: The use of new materials has improved the drainage system of streets and alleys, also added vegetation space.

Much of the road network in Meizhou’s historical area is of old origin and significantly deviates from current modern municipal construction standards. The roads are generally narrow and in poor condition. During planning and renovation, the transportation layout was reorganized and dedicated parking spaces were designated, thereby freeing up previously occupied alley areas from temporary parking use. In Fig 15, the occupied outdoor platform will be transformed into a “sponge platform” with water-absorbing and green vegetation functions, improving both pedestrian comfort and urban green space. Additionally, the quality of slow traffic zones on sidewalks will be enhanced, making the road drainage system more efficient and adaptable.

**Fig 16:**
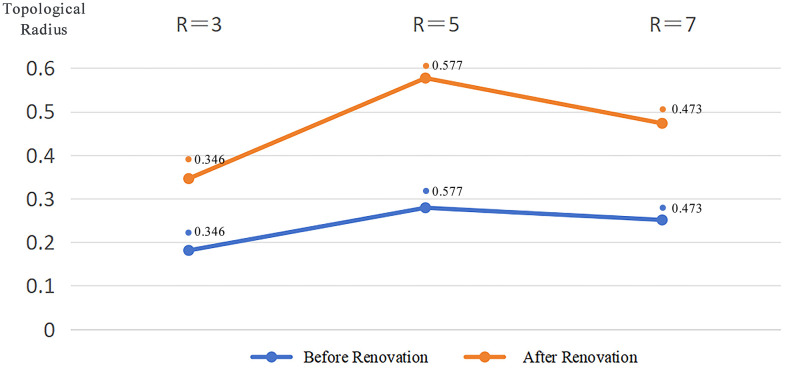
Comparison of symbiosis values before and after renovation.

Based on the aforementioned renovation suggestions, a systematic optimization and renovation in the public spaces of Meizhou Ancient City have been carried out. By recalculating the axial model post-renovation using Depthmap software, we obtained detailed data models for each spatial unit after the renovation. The comparison of data before and after the renovation revealed the following significant changes:

Firstly, the symbiosis value post-renovation has significantly increased, showing an upward trend as the topological radius range expands. This clearly demonstrated that the ancient city’s capacity to support various functions has been significantly enhanced, contributing to increased functional diversity. This change optimized the adaptability and resilience of the space in Fig 16.

**Fig 17:**
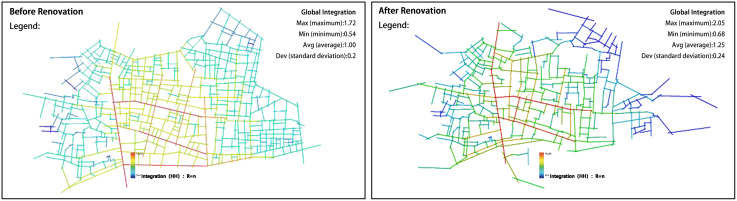
Analysis and comparison of global integration.

Secondly, the global integration values of the main streets have generally increased after the renovation, reflecting a significant improvement in the connectivity of the internal public spaces in Fig 17. This change indicated an enhanced accessibility within the city’s internal road network, leading to a more dynamic and efficient flow and interaction of elements within the spatial framework. Consequently, this change greatly strengthened the anti-interference capability and stability of the ancient city’s public space in Table 3.

**Table 3:** Analysis of global integration of main roads.

Road Name	Lingfeng East Road	Lingfeng West Road	Zhongyuan East Road	Zhongshan Road	Jinshan Road
Global Integration Before Renovation	1.20	1.50	1.68	1.72	1.10
Global Integration After Renovation	1.55	1.94	1.78	2.00	1.43

Finally, the renovation of Meizhou Ancient City demonstrates improved structural resilience, with enhanced integration of the main street network (Fig 16), leading to more efficient crowd evacuation and reduced congestion during peak tourism periods. Functional resilience has also been strengthened, as indicated by increased symbiotic value (Fig 17), enabling the space to flexibly accommodate commercial, residential, and community service functions and better respond to unexpected demands such as public health emergencies. Additionally, cognitive resilience has improved through interface continuity design (Table 3), with spatial comprehensibility rising from R^2^ = 0.23 to 0.54, effectively reducing way finding difficulties for both residents and visitors.

In summary, the proposed renovation strategies have enhanced the adaptability, resilience, and flexibility of public spaces in the Meizhou historic district. By improving connectivity, optimizing the street network, and refining the spatial layout, these strategies have collectively strengthened the district’s overall spatial resilience.The improvement across various indicators suggested that the proposed strategies are both practical and effective, effectively supporting the sustainable development of public spaces in historic cities.

## 6. Conclusions

### 6.1. Application

Traditional urban planning often addressed singular functional needs of their time, resulting in many historic towns struggling to accommodate diverse contemporary demands [11]. Inefficient spatial use and low functional adaptability are now common. Moreover, the need to balance preservation with development further constrains urban renewal efforts [27]. Many renovated spaces remain underused and economically inflexible, failing to align with modern societal dynamics.

Space syntax offers a robust framework for analyzing the evolving public spaces of historic towns. By integrating syntactic measures, it quantitatively assesses spatial structure and correlates it with current public behavior patterns [28]. This approach provides an objective foundation for sustainable spatial design, enhancing both scientific validity and practical feasibility in urban conservation and adaptation.

### 6.2. Strategy

This study applies space syntax analysis to evaluate public spaces within Meizhou’s historic urban core, developing a syntactical framework to assess spatial performance and propose integrated revitalization strategies for its mixed-use commercial and residential districts. The proposed strategies emphasize sustainability through historical continuity, functional adaptability, and community involvement. Specific measures include reinventing existing spaces, diversifying spatial uses, and enhancing street-level commercial interfaces. Space syntax validates the effectiveness and feasibility of these interventions. Furthermore, as an evaluative tool, it supports the continuous refinement of activated historic public spaces post-regeneration.

### 6.3. Universal applicability

By quantifying the relationship between spatial structure and function in Meizhou’s historic urban area through syntactic measures such as integration, mean depth, and clustering coefficient, this study enables accurate evaluation of functional mix, street connectivity, and the safety of open spaces—key dimensions influencing spatial resilience. Based on these insights, strategies like “adaptive reuse of existing structures” and “shared street space” are proposed, demonstrating strong regional applicability. The quantitative framework and implementation pathway established here offer methodological support for the sustainable regeneration of similar historic urban districts.

Given the spatial structure and cultural characteristics of Meizhou, a typical historical city in Lingnan region, the strategies proposed in this study have the potential to be applied to other small-scale historical urban areas. However, it must be emphasized that the effectiveness of any sustainable regeneration strategy is highly dependent on a deep understanding of the local spatial layout, social needs, and cultural context. As revealed by spatial syntax – high resilience does not come from a universal template, but stems from respecting and activating the local structural logic. Therefore, future practices should build on the quantitative tools provided in this study and, in combination with local actual conditions, achieve the coordinated evolution of protection and renewal.

### 6.4. Limitation

As an innovative quantitative tool, space syntax has been increasingly applied in urban planning, offering new perspectives and methods for the sustainable design of public spaces in historic cities. However, the physical and social spatial relationships in such contexts are often complex. In the case of Meizhou—a historic urban area, though not ancient in the conventional sense—data collection posed significant challenges, including incomplete documentation of traditional architecture, scarce records of socio-spatial relations, and fragmented historical evolution data. Furthermore, extensive unauthorized modifications within the urban fabric have obscured the original spatial structure, compromising the accuracy of the syntactic model and the subsequent resilience analysis, thus leading to potential omissions and inaccuracies. Despite thorough efforts, the exploratory nature of this methodology and the author’s own scholarly limitations suggest that, while contributing innovative insights, the study requires further refinement.
